# Supplementary material for: Integrated analysis of the aqueous humor microbiome and lens capsule transcriptome in high myopia cataract: a pilot study
Source: Front Med (Lausanne). 2026 Jun 16;13:1845205. doi: 10.3389/fmed.2026.1845205 (PMC13314463; doi:10.3389/fmed.2026.1845205)
Supplement: Supplementary file 3 [file Data_Sheet_1.zip › 7.Average/1.Community_Structure/heatmap/C372089/Species_top15_nocluster.pdf]

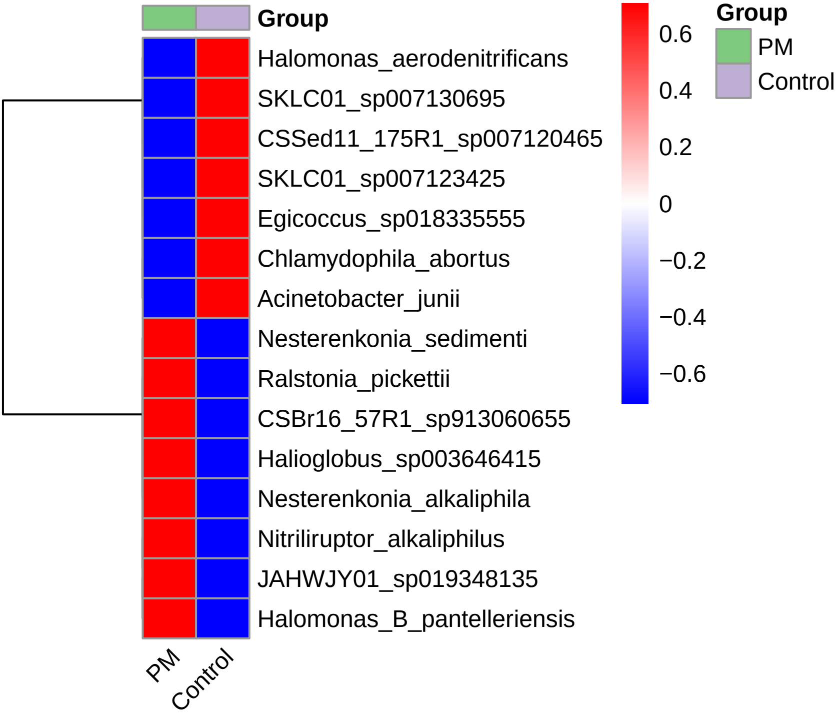
Group
Halomonas_aerodenitrificans
SKLC01_sp007130695
CSSed11_175R1_sp007120465
SKLC01_sp007123425
Egicoccus_sp018335555
Chlamydophila_abortus
Acinetobacter_junii
Nesterenkonia_sedimenti
Ralstonia_pickettii
CSBr16_57R1_sp913060655
Halioglobus_sp003646415
Nesterenkonia_alkaliphila
Nitriliruptor_alkaliphilus
JAHWJY01_sp019348135
Halomonas_B_pantelleriensis
PM
Control
0.6
0.4
0.2
0
−0.2
−0.4
−0.6
Group
PM
Control
